# Using spatial frequency domain imaging to monitor a skin biopsy wound: a pilot study

**DOI:** 10.1364/BOE.536843

**Published:** 2024-09-13

**Authors:** Lai Zhang, Alistair Bounds, John Girkin

**Affiliations:** 1 Centre for Advanced Instrumentation, Department of Physics, Durham University, Durham DH1 3LE, United Kingdom; 2 Occuity Ltd, The Blade, Abbey Square, Reading RG1 3BE, United Kingdom

## Abstract

Surgical wound infection is a global postoperative issue adding a significant clinical burden and increasing healthcare costs. Early detection and subsequent diagnosis of infection is vital for accurate, early, and effective treatments. In this paper, we report a pilot study exploring spatial frequency domain imaging (SFDI) to monitor, *in-vivo*, a biopsy wound in human skin. The reduced scattering coefficient, $\mu_{s}^{\prime }$ , absorption coefficient, $\mu_{a}$ and the oxygen saturation, *StO*_2_, were measured using a SFDI system at 617 and 850 nm. We found the $\mu_{s}^{\prime }$ was better capable of monitoring structural changes, possible pus within the wound, re-epithelialization, and collagen fiber remodeling, than with the eye alone. The $\mu_{a}$ map is capable of revealing the total hemoglobin distribution in the wound area but was limited in some regions due to the scab covering. This case study indicates SFDI’s potential for monitoring and quantifying the process of surgical wound healing and infection.

## 1. Introduction

In the UK, surgical site infections (SSI) account for up to one in seven hospital patient acquired infections [1]. Patients with SSI are required to stay on average 7 - 11 days longer than patients without such wound infections. Currently 98% of wound infections are detected using visual inspection and patient-reported symptoms at a point where significant clinical work is required to resolve the infection [2–4]. Since infection is acknowledged as an impediment in wound healing [5], early diagnose is clinically vital as well as relieving a patient’s distress and discomfort during surgical recovery. The gold standard [6,7] for diagnosis is through a wound culture developed from a swab or biopsy. These methods are invasive and take several days to culture, frequently making it too late for early treatment. A non-invasive and early diagnosis method is urgently needed to observe the wound healing process including structural changes and indicators for metabolic changes and possible infection signs. To enable such diagnosis and aid with a worldwide health care problem, an imaging method is required that is cost-effective, simple to operate and rapid.

Non-contact imaging techniques can provide greater information than the naked eye [8]. Though previous research works applied to wound imaging were not specifically to investigate post-surgical wound cases, they may have potential for use in this application. Optical coherence tomography (OCT) uses the optical scattering properties of the tissue via point-by-point scanning imaging with a spatial resolution of between 1-15 *μ*m. [9] It has been used to monitor tissue structural changes including burn wound depth [10], epidermis migration [11–13] and collagen denaturation [14]. However, scanning time is relatively long and OCT cannot measure the chromophores in the wound. Hyperspectral imaging (HSI) [15–17] has been applied to assess tissue pathophysiology including open wounds [18], diabetic foot ulceration [19,20], burn wounds [21] and skin perfusion [22,23]. The main biological tissue indicators for hyperspectral wound imaging include water, hemoglobin, oxygenation map and melanin concentration which can be extracted from the reflectance data cube [24]. However, HSI instrument is very expensive [25] to build which adds a significant barrier for the potential users and it is not available to measure the structural information of the skin. Laser Doppler imaging (LDI) [26,27] is capable of measuring the blood perfusion for burn wounds [28,29] based on the Doppler effect. However, LDI cannot provide structure information and chromophore concentration in the tissue. Optoacoustic imaging (OA) is advanced in detecting the chromophores through the human skin and reconstructing capillary and lipid structures with high resolution (laterally 4 *μ*m) based on the photoacoustic effect [30,31]. It has been applied to detect and quantify burn wound depth [32–34], burn collagen fiber anisotropy with polarization and ulcers. [35]. However, the field of view is limited to a couple of millimeters [36] while the scanning time is relatively long.

Spatial frequency domain imaging (SFDI) is a non-contact, rapid and wide field of view skin imaging technique [37,38]. It has been applied in the assessment of burn wounds, initially with porcine and rat models [39–41], diabetic foot ulcers [42], pressure ulcers [43] and scleroderma [44]. It can indicate the structural change via reduced scattering coefficient measurements whilst map absorption coefficients for the chromophore concentration in the wound. It is a cheap instrument to build costing around $2000 [45]. The trade off is the 1 mm spatial resolution which is still significantly small compared to the size of the surgical wound which is typically a few centimeters in the length. Therefore, the SFDI method here matches our purpose best to explore surgical wound diagnosis.

During the SFDI imaging process, three phases of a sinusoidal illumination pattern are projected separately onto the tissue. By capturing the returned diffuse reflectance, the reduced scattering coefficient, $\mu_{s}^{\prime }$ , and the absorption coefficient, $\mu_{a}$ , can be calculated for the target area. SFDI has not only been explored for use in clinical assessment for wound and skin complications SFDI is also capable of detecting early signatures of disease, for example, detecting demineralization in dental enamel prior to dental caries [46], and endoscopic screening for gastrointestinal cancers [47].

However, these previous SFDI applications typically assume the tissue has a homogeneous horizontal structure. SFDI has not previously been extended to surgical wound site monitoring which have a specific heterogeneous vertical structure due to the surgical incision. There has been research discussing SFDI’s spatial resolution in vertical structures for breast cancer margins [48] and tumor resection [49,50]. SFDI has been proved numerically to characterize a vertical heterogeneous structure in wounds [51–54].

In this paper, we firstly review the wound healing knowledge and the protocol for wound measuring. The reduced scattering and absorption result from SFDI are analyzed with the VIS image of the wound.

## 2. Wound healing process and infection

**Fig. 1.**
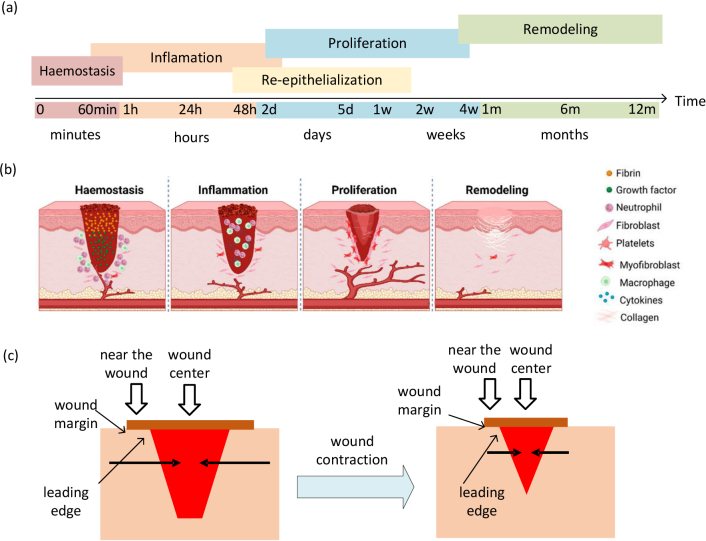
(a) Timeline for the four sequential and overlapping wound healing phases. [56–59]. (b) Four phases of the wound healing and their mechanisms. [60] (c) Detailed wound contraction process during the proliferation phase [61]: the wound contraction happens from the edge to the center shown by horizontal arrows. The red block represents the wound area and the brown block presents the wound margin can be seen from the surface.

There are four overlapping phases [55,56] in the wound healing process: early response, inflammation, proliferation and remodeling. A diagram demonstrating the physiological process is shown in Fig. 1.

**Early response:** Hemostasis appears immediately when blood starts to leak [58]. The blood clot is formed from insoluble fibrin which fills the wound bed as a provisional wound matrix.

**Inflammation:** Immediately after hemostasis, the injured blood vessels leak transudates leading to local swelling which appears as inflammation. The soluble fragment of the degraded collagen recruits immune cells whilst acting as a signal to promote the development of new blood vessels [56,62]. Visually, a scab is formed to protect the wound and provide an ideal moisture rich environment for wound healing.

**Proliferation:** Here, granulation tissue replaces the fibrin clot in the wound with capillary-rich fibroblastic tissue [63,64] by fibroblast synthesis and collagen production. As a result, the wound contracts due to the granulated tissue gradually filling the wound gap [65] from the edge to the center as shown in Fig. 1(b). In parallel with this, the re-epithelialization takes place where keratinocytes migrate from the edge to the center of the wound.

**Remodeling:** In this phase, the new epithelium and final scar are developed. The capillaries stop growing and blood flow to the wound area decreases [66,67]. The collagen matrix in the dermis is remodeled from type 3 to type 1 [62,68] forming a more stable collagen structure and then the tissue increases in tensile strength.

## 3. Method

### 3.1 Wound information and protocol

A 57-year-old male underwent an incision biopsy on his right hand due to a suspicion of skin cancer. The biopsy wound was closed with non-absorbable sutures after the lesion had been sampled. The surgery took place 14 days before we started the imaging experiment. We undertook a 42 day observation of the volunteer’s wound healing progress until his wound was visibly healed for 3 weeks. The wound was suspected of becoming infected on day 0 of our observation and our volunteer was prescribed oral antibiotics for 5 days.

**Fig. 2.**
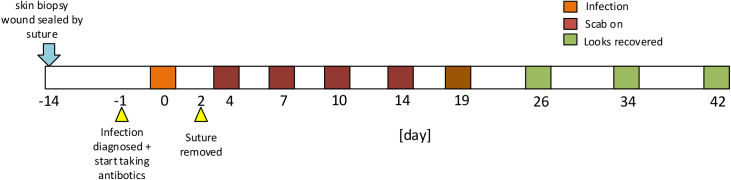
Timeline for the wound healing and monitoring. The wound was monitored every three or four days before the scab came off as the wound condition changed. The healed wound area was then imaged with a seven day interval as the tissue reformation phase takes place more slowly.

We wished to observe the structural changes in the wound through the reduced scattering coefficient, $\mu_{s}^{\prime }$ , and hemodynamic profiles via the absorption coefficient, $\mu_{a}$ . The timeline for the surgery and the SFDI monitoring is shown in Fig. 2. The protocol in this paper was approved by the Ethics Committee of the Department of Physics in Durham University. The wound area and the same region on left hand were imaged at each visit as a reference and calibration for daily fluctuations in skin condition [69]. Two wavelengths were selected at 617 nm and 850 nm to assess wound optical properties at different depths (longer wavelength can reach deeper) and to measure the relative ratio of deoxy-hemoglobin (Hb) and oxy-hemoglobin (HbO_2_) in order to calculate the oxygen saturation (StO_2_) [70]. The $\mu_{a}$ value at 617 nm indicates the total hemoglobin concentration.

### 3.2 Instrumentation

**Fig. 3.**
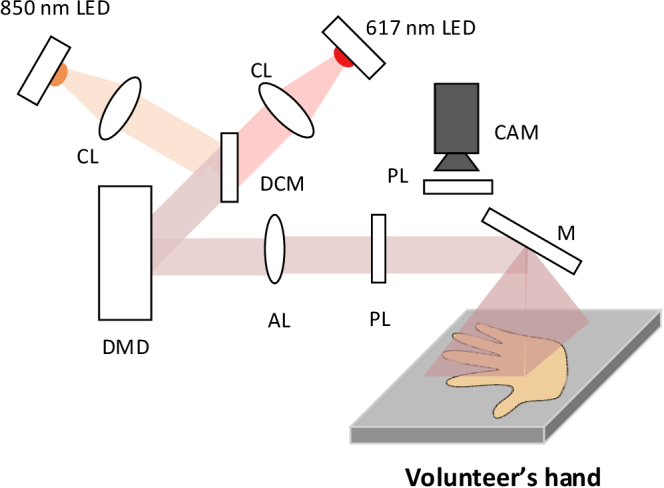
The geometry using dual wavelength LEDs to image the biopsy wound on volunteer’s hand. CL = collimating lens focal length 16 mm, DCM = dichroic mirrors, AL = achromatic lens focal length 50 mm, PL = polarizer, M = mirror, CAM = camera (with 35 mm focal length lens).

The geometry of the SFDI system follows the openSFDI design [45] and is shown in Fig. 3. Here, two LEDs at 617 nm (Thorlabs, M617D2) and 850 nm (Thorlabs, M850D2) are utilized for the illumination. The digital mirror device (DMD) encoded the collimated beam from the LEDs with sinusoidal pattern and then an achromatic lens (focal length 50 mm) was used to magnify the pattern from the DMD on to the skin. To enable the two wavelengths to be projected onto the same area of skin a dichroic mirror was placed between two the LEDs to reflect the 850 nm light beam on to DMD, whilst passing the 617 nm light beam through in the original direction. Two orthogonally aligned polarizers were placed between the focusing lens and camera to eliminate the surface reflection from the skin.

Three phases of the sinusoidal pattern were sequentially projected onto the skin surface with relative phases 0, $\frac{2\pi }{3}$ and $\frac{4\pi }{3}$ rad. A USB camera (BFS-U3-13Y3M-C, Blackfly Camera, Edmund Scientific) collected the diffuse reflectance image with a 35 mm lens. A spatial frequency of 0.1 mm^−1^ was used to give sufficient penetration depth to evaluate the wound underneath the epidermis. (The estimated penetration depth of the sine pattern is 1.38 mm at 617 nm and 1.57 mm at 850 nm for the healthy skin area.) To calibrate the system for the scattering coefficients a sample was made with epoxy resin and TiO_2_ powder with the optical properties of reduced scattering coefficient $\mu_{s}^{\prime }=1.2\;{\text{mm}}^{-1}$ , absorption coefficient $\mu_{a}=0.004\;{\text{mm}}^{-1}$ at 617 nm and reduced scattering coefficient $\mu_{s}^{\prime }=0.08\;{\text{mm}}^{-1}$ , absorption coefficient $\mu_{a}=0.004\;{\text{mm}}^{-1}$ at 850 nm [51].

### 3.3 Data processing

The alternating component (AC) image is obtained with Eq. (1) [37],


(1) $$I_{AC}=\frac{\sqrt{2}}{3}\sqrt{{\left(I_{1}-I_{2}\right)}^{2}+{\left(I_{1}-I_{3}\right)}^{2}+{\left(I_{2}-I_{3}\right)}^{2}}$$

where $I_{1}$ , $I_{2}$ and $I_{3}$ are the three-phase diffuse reflectance images. The AC images were first binned with a 5 × 5 window to reduce the noise whilst maintaining the required spatial details of the wound. Then a Scale-Invariant Feature Transform (SIFT) [71] and a Random Sample Consensus (RANSAC) [72] were applied to remove any motion artifacts in the AC images during the dual wavelength measurement. The absorption and reduced scattering maps were recovered from the registered AC images with the look-up-table method provided by appSFDI [73] software.

## 4. Results

### 4.1 Infection observation

**Fig. 4.**
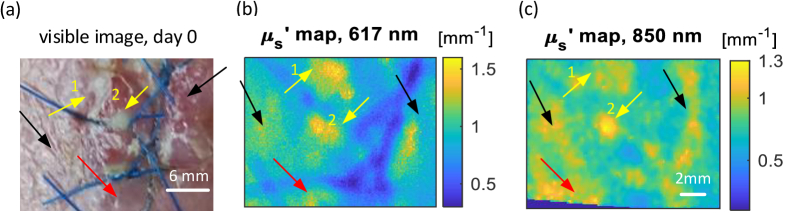
Visible image and scattering maps at day 0 (a) The visible image of the wound, day 0. (b) The reduced scattering $\mu_{s}^{\prime }$ map at 617 nm for the wound at day 0. (c) The reduced scattering $\mu_{s}^{\prime }$ map at 850 nm for the wound at day 0. The red arrows show the potential area with pus, which is hard to see in the conventional visible image but can be seen from the $\mu_{s}^{\prime }$ maps. The yellow arrows show the pus indicated by the high reduced scattering coefficients and just visible in the conventional image. The black arrows point out the area believed to have pus pooling more deeply in the tissue clearly seen in the 850 nm $\mu_{s}^{\prime }$ map but hard to notice in the 617 nm $\mu_{s}^{\prime }$ map and visible image.

The biopsy wound was suspected of becoming infected at day 0 of the observation. As illustrated in Fig. 4(a), the wound site image shows the pus as white spots with additional redness near the suture sites. In Fig. 4(b) and (c), the pus sites demonstrate a higher $\mu_{s}^{\prime }$ value at both wavelengths. However, the redness around the wound had no significant effect on the $\mu_{s}^{\prime }$ map indicating that the $\mu_{s}^{\prime }$ is not sensitive to the blood flow and vessel changes which clinically cause the redness in the image. This indicates the $\mu_{s}^{\prime }$ is capable of detecting the structural changes caused by the pus, a by-product of body’s defense mechanisms against infection.

As indicated by the red arrows, pus can be seen pooling with a higher value in both $\mu_{s}^{\prime }$ maps but only showing redness in the conventional visible image. Comparing the areas indicated by the yellow arrows highlights the pus sites in the $\mu_{s}^{\prime }$ maps and visible image, with the area of the pus always appearing larger in the $\mu_{s}^{\prime }$ maps than in the visible image, i.e. the condition of infection was more severe than the naked eye could see due to the features hidden beneath the surface. Looking closely, sites 1 and 2 have similar $\mu_{s}^{\prime }$ values at 617 nm and intensities in the visible image. However, in the 850 nm $\mu_{s}^{\prime }$ map, site 1 has a lower $\mu_{s}^{\prime }$ value than site 2. The reason behind this may be that site 2 had formed a deeper pus cavity and hence a more severe infection condition than site 1. The 850 nm wavelength penetrates more deeply and can thus see pus forming at a greater depth than that seen by the 617 nm. Such a difference is illustrated by the black arrows.

**Fig. 5.**
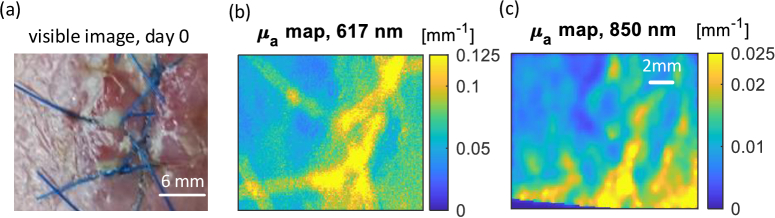
Visible image and absorption maps at day 0 (a) The visible image of wound area, day 0. (b) The absorption $\mu_{a}$ map at 617 nm for the wound at day 0. (c) The absorption $\mu_{a}$ map at 850 nm for the wound at day 0. The sutures have different absorption coefficients at the two wavelength.

Comparing $\mu_{a}$ maps in the Fig. 5 with $\mu_{s}^{\prime }$ maps in the Fig. 4, the difference in the healthy skin and infection site is not evident in $\mu_{a}$ . The $\mu_{s}^{\prime }$ is a promising indicator for structural change due to the pus formation, whilst the $\mu_{a}$ maps are not sensitive to the pus at the two wavelengths used.

### 4.2 Physiological observation

**Fig. 6.**
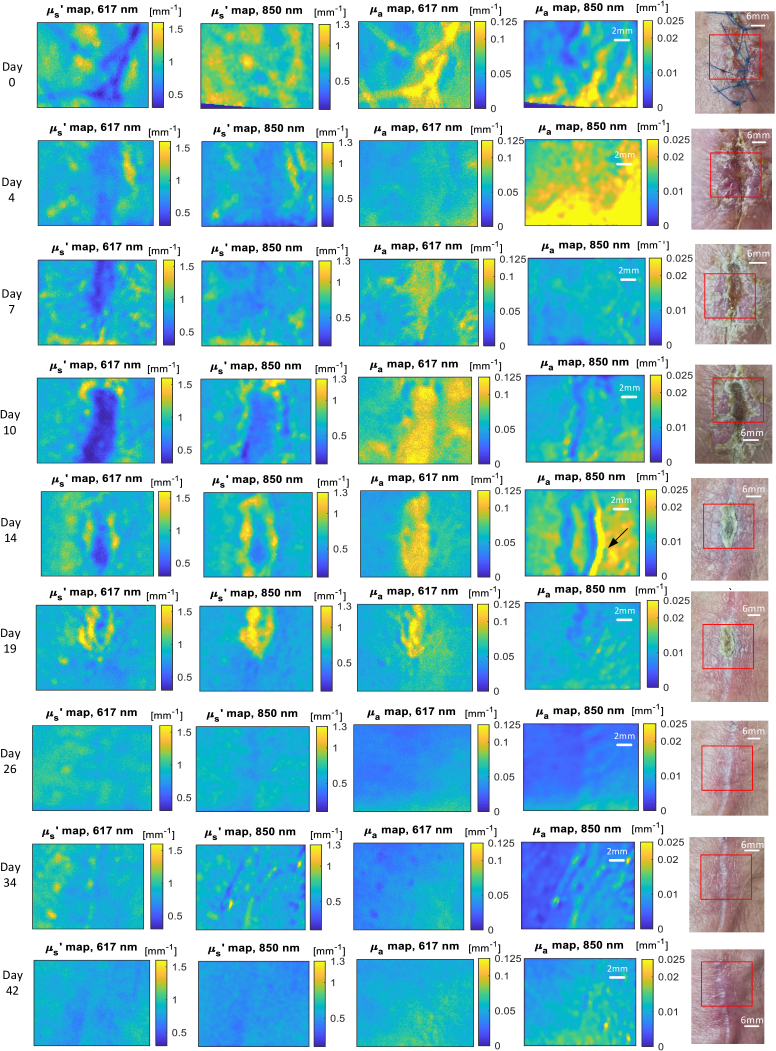
The wound healing images from day 0 to day 42. Each row is for a specific day and each column is for one optical property or visible image. The first to fourth columns from the left present the recovered optical properties map at the two wavelength.The right most column is the visible picture for the entire area of the wound site and the red rectangle indicates the area shown in the optical property maps.

The $\mu_{a}$ maps, $\mu_{s}^{\prime }$ maps and visible images for the whole period of observation are shown in Fig. 6. On day 0 and day 4, uneven hand placement, minor motion or the hand curvature was likely to add uncertainty to the absorption maps at 850 nm. This effect is readily apparent as the absorption coefficient value at 850 nm are significant lower. From the visible images, at day 4, the wound area is red in appearance. This indicates the capillaries were forming during the dermal replacement. From day 4 to day 7, the wound site demonstrates a higher value in $\mu_{a}$ possibly due to scab formation combined with the expected blood volume increase. The scab gets thicker from day 7 to day 10 shown in both the visible picture and a higher value in the 617 nm absorption maps. The 850 nm may get through the scab or it is not sensitive to the wound tissue below the scab compared to the surrounding skin. The low $\mu_{s}^{\prime }$ value in the wound area can be explained by the expected dissolution of the collagen fibers during the wound healing process. The edge of the wound area always has a high reduced scattering probably due to re-epithelialization. The low scattering area shrinks day by day, showing the wound was healing and contracting gradually in days 4 to 19.

On day 14, the absorption map at 617 nm clearly shows the extent of the scab whilst at 850 nm there is a high absorption line at the right of the wound as indicated by the arrow. We believe this is an artifact due to the detachment of the skin and the angle of imaging at this point. When the scab detaches at the edge, the incident light "bounces" several times in the thin air layer between the scab and skin and is lost to the detector. The absorption coefficient therefore has a higher value in this area.

In these early stages of wound healing SFDI appears to detect the changes in the wound beneath the scab and changes in the scab providing a monitor of the wound healing process better than the naked eye. When the scab fell from the wound, from day 19, the regeneration was still ongoing. The high scattering intensity spots in day 26 and day 34 may be caused by the suture and wound scar formation. This indicates the collagen fibers are growing and transforming in type so again SFDI is providing greater information on wound progression then the visible image alone. Further details will be discussed in the following section.

### 4.3 Optical properties observation

**Fig. 7.**
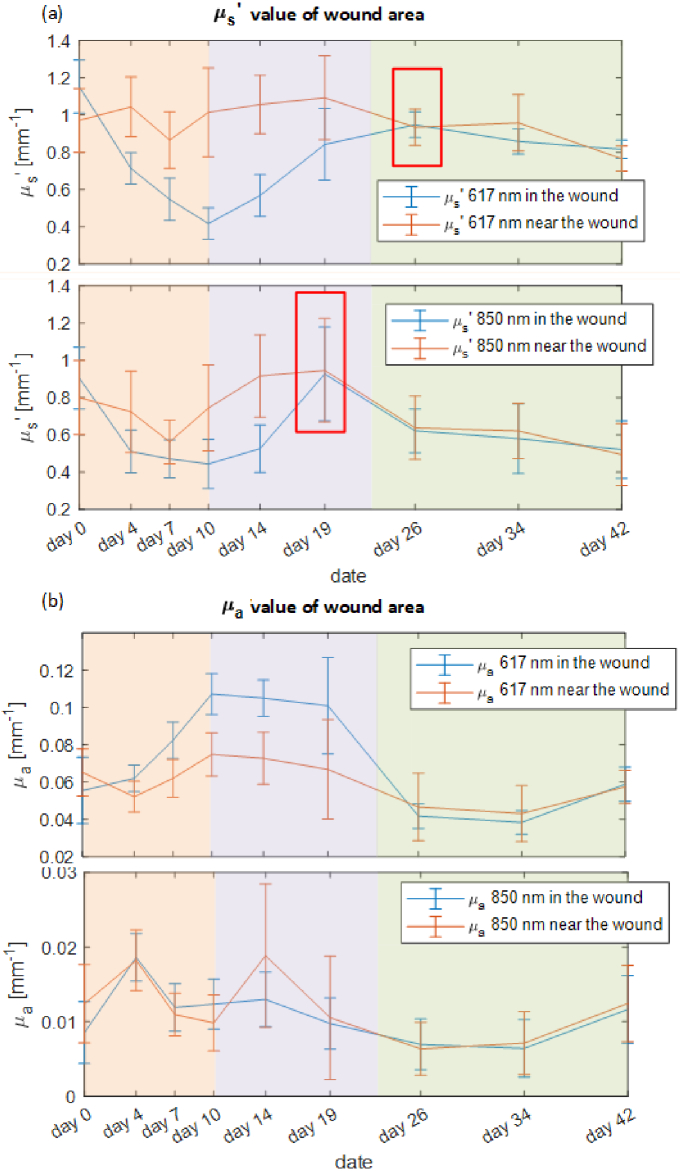
(a) The reduced scattering coefficient curves for wound and area near the wound at 617 nm and 850 nm separately. The red rectangles indicate the point two curves start to overlapped to each other. (b) The absorption coefficient curves for wound and area near the wound at 617 nm and 850 nm separately.

The wound area is determined by the relatively low scattering value on the $\mu_{s}^{\prime }$ map. To verify the wound healing model, the area near the wound margin is also selected, with approximately a 1 mm margin as shown in Fig. 1(b). The mean value of the reduced scattering and absorption of two wavelengths are calculated and demonstrated in Fig. 7 with the error bar obtained from the standard deviation of all pixels within the wound or margin area.. In the absorption curves, the 617 nm is more insightful to the chromophore change in the wound healing process than the 850 nm.

From day 0 to day 10 (see orange area), collagen fiber is transferred into the fibroblasts gradually, forming the granulation tissue to replace the provisional wound matrix. This caused a fall in the reduced scattering coefficient in the wound center as well as the area near the wound. A similar $\mu_{s}^{\prime }$ decreasing phenomena, due to the granulation tissue, was also observed in the burn wound [74,75]. With the granulation tissue gradually forming within the wound, blood vessels contained within the wound result in an increased blood content both in and near the wound, driving a rise in absorption in the 617 nm curves of both wound and, to a slightly lesser extend, near the wound as shown in the top graph in Fig. 7.

In the time range indicated by the purple shading, the $\mu_{s}^{\prime }$ curves in both wavelengths of Fig. 7(b) follow a rising trend and finally become overlapped until the end of observation. This may indicate the granulation tissue altering from homogeneous to heterogeneous where the fibroblasts laid down into collagen fibers to stabilize and contract the wound. The $\mu_{a}$ value stayed stable during this period probably due to the scab was fully formed and dominated the absorption recovered from the wound. According to our wound healing knowledge, the vascular network might be continuing to be restored under the scab in the angiogenesis process. The scab came off after day 19 when the wound was visibly healed and switched into remodeling stage. The $\mu_{a}$ values at 617 nm then dropped for both wound areas comparing to day 10 as the newly generated vessel decline.

Notably, the wound contraction was ongoing simultaneously from day 0 to day 26 as demonstrated by the distance between the wound and area near the wound’s optical properties curve. In the structural aspect, the area near the wound had greater $\mu_{s}^{\prime }$ value than in the wound center. Also, the $\mu_{s}^{\prime }$ curves in 617 nm graphs began to be overlapped from day 26 whilst there at 850 nm (where photon penetration depth is deeper) begin to overlap at day 17 (see red rectangles in Fig. 7(b)). These indicate the wound contracting from the bottom to top and edge to center. Additionally, there is a significantly higher level of absorption in wound than the area near the wound. This suggests that center of the wound requires more blood supply, or that the edge or the blood vessels, decline earlier than in the wound center. Both $\mu_{s}^{\prime }$ and $\mu_{a}$ curve trends reflect the wound healing V-shaped model as shown in Fig. 1(b).

### 4.4 Hemodynamic observation

Oxygen supply plays a vital role in the success of wound healing. The numerous biological processes demand an adequate oxygen supply and appropriate oxygen levels also triggers the tissue healing response [76,77]. The blood supply for oxygen delivery will be greatest at the wound margin.

**Fig. 8.**
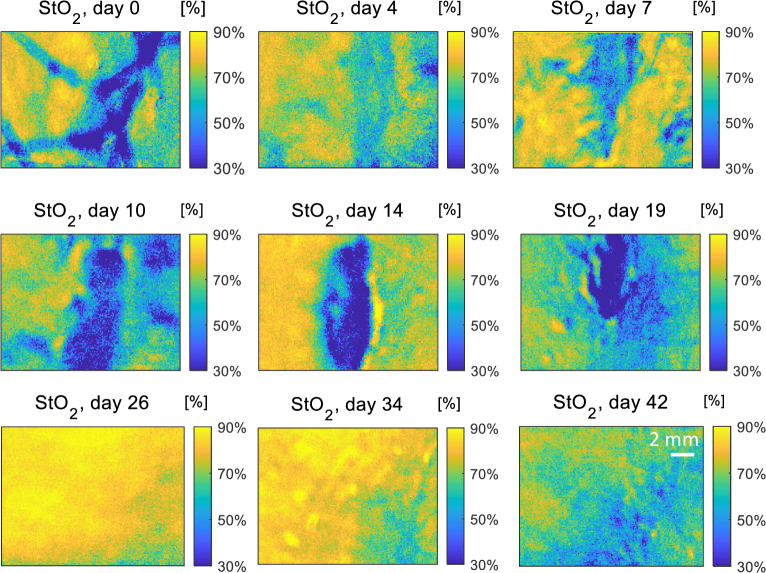
The oxygen saturation maps for the wound from day 0 to day 42.

The maps for oxygen saturation (StO_2_) are shown in Fig. 8. On day 0, the saturated oxygen level at the infection site is very high, indicating the large oxygen requirement of immune cells defending the body against bacteria. The wound area near the sutures is relatively low in oxygen saturation matching the hypoxia expected in the inflammation phase. This is potentially also an indicator of a delay in wound healing.

From day 4 to day 7, the area around the wound has relatively high oxygen saturation as the wound healing requires adequate blood supply from outside the wound. In days 10 to 19, the scab has fully formed and became thick with strong absorption that blocks the sinusoidal pattern from being transmitted and backscattered from deeper within the wound. Due to the scab the absorption map here is not reliable enough for quantifying the wound, leading to an unexpectedly low value StO_2_ at scab area. There is expected to be from tissue proliferation and vessel rebuilding based on our wound healing knowledge but we do not have evidence of this as this is hidden beneath the absorbing scab. From the area surrounding the wound, temporary hypoxia occurred again to stimulate the growth factor [76] as an initiator for the angiogenic growth and re-epithelialization. On day 14, as predicted by the $\mu_{s}^{\prime }$ maps, wound contraction is occurring and increasing the demand on oxygen indicated by the high oxygen level in the area surrounding the wound.

After the wound is visibly healed, moving into the final wound healing phase, the oxygen saturation is high as observed on day 26 and day 34 when the wound is in a remodeling phase. At this time, the type 3 collagen is being replaced by type 1 which is dependent on sufficient oxygen for the transformation to take place [76]. When the collagen replacement is complete, the oxygen saturation fell to the normal level.

## 5. Conclusion and discussion

For the first time we have monitored a typical surgical biopsy wound with the SFDI technique. SFDI clearly aided in detecting the infection at an early stage and provided information on the wound healing progress. Reduced scattering coefficient provides an indication of the structural changes taking place in the tissue including the formation of pus and collagen fibers. Damage to the collagen fibers leads to a lower $\mu_{s}^{\prime }$ value matching the result from previous work [78]. The pus collecting within the cavity produced a high scattering value indicative of the high density of the dead and diseased tissue inside. Applying the dual wavelength measurement, the V-shaped wound healing model is observed through the different depth measurements. We observe the wound contraction via the $\mu_{s}^{\prime }$ and the blood volume change by the $\mu_{a}$ . The time series StO_2_ calculated using the two wavelength values of $\mu_{a}$ serves as another prospective bio-marker for monitoring the wound healing process.

The $\mu_{s}^{\prime }$ can penetrate through the scab to present the wound structural changes underneath. However, recovery of the $\mu_{a}$ fails through the scab tissue. The reason here might be that in the biological tissue the $\mu_{s}^{\prime }$ is far greater than the $\mu_{a}$ . Thus, $\mu_{a}$ is more difficult to measure accurately.

Previous research [51] has demonstrated that the the difference between the $\mu_{s}^{\prime }$ in the neighboring structures the easier it is to detect the difference. This matches our observation that pus at day 0 demonstrates a good contrast with double the $\mu_{s}^{\prime }$ value of the surrounding skin. Here the partial volume effect [79] exists in that SFDI overestimates the wound width in a vertical structured wound. The wound area from the $\mu_{s}^{\prime }$ map probably has a smaller size than indicated by lower $\mu_{s}^{\prime }$ value, especially for the days 14 to 19 (see Fig. 6). However, $\mu_{s}^{\prime }$ here still presents the real size of the wound more accurately than the naked eye. Longer wavelengths demonstrate a smaller wound site underneath the scab validating a V-shaped 3D wound structure. Our observations thus fit with the current physiological model of wound healing.

## 6. Future work

We report a single case of an infected wound observation after the wound had been forming, for 2 weeks. In the future, we are looking to have animal models with vertical surgical wounds and creating the infection with bacteria [41]. This will help us to control the infection and the initial condition of the wound. We will also measure the wound more closely at the day from creation of the wound. We will also look to build an instrument suitable for use in the clinic. This will help us to build a database of human wound healing parameters when measured using SFDI, from which the earliest signs of infection may be detectable prior to when they could be detected through visual inspection alone.

In our case, the pus, collagen fiber and epidermis changes are detected in the wound tissue. In terms of structure, we are also keen to observe the scar formation after the wound is visibly healed. Polarization will be integrated into the current system for better monitoring of the scar and to help increase the contrast from surface reflections from the tissue [80,81]. We would also look more deeply beneath the scab to see the wound healing features. For this purpose, we may optimize the wavelength used in the SFDI system to have greater penetration under the scab. To apply SFDI in other body areas, especially areas with lower optical properties values, structure curvature needs to be taken into account to obtain accurate results. [82]. One may consider a 3D shape measurement combined with the SFDI measurement based upon the shape of the reflected line combined with the SFDI measurement. This could be achieved by adjusting the polarizing filters such that the reflected light is detected rather than rejected in the current configuration.

SFDI wound monitoring may also be combined with other optical imaging techniques, such as hyperspectral imaging [24,83,84] helping to separate the melanin distribution and laser Doppler imaging [85,86] for blood flow change.

## Data Availability

Data underlying the results presented in this paper can be be obtained from the authors upon request.

## Appendix

The relative oxygen saturation $\left(StO_{2}\right)$ is calculated based on the absorption of the extracted $\mu_{a}$ in two wavelength.

(2)
$$\mu_{a}(\lambda )=ln(10)[\varepsilon_{HbO_{2}}(\lambda )c_{HbO_{2}}+\varepsilon_{Hb}(\lambda )c_{Hb}]+\mu_{a,water}(\lambda )\cdot 70\%$$

The $HbO_{2}$ is the oxy-hemoglobin and the Hb is the deoxy-hemoglobin. The molar extinction coefficients are $\varepsilon_{HbO_{2}}(\lambda )$ and $\varepsilon_{Hb}(\lambda )$ correspondingly. $c_{HbO_{2}}$ is the molar concentration of $HbO_{2}$ and $c_{Hb}$ is the $c_{Hb}$ of Hb . The $\mu_{a,water}$ is the absorption of the water at the wavelength λ assuming the water content of human skin is 70%. The oxygen saturation $StO_{2}$ is calculated with

(3)
$$StO_{2}=c_{HbO_{2}}/(c_{HbO_{2}}+c_{Hb})$$

Here the molar extinction coefficients for the two wavelengths are listed in the Table 1 from the omlc website [87].

**Table 1.** The molar extinction coefficients for $HbO_{2}$ and Hb .

Wavelength	$\varepsilon_{HbO_{2}}/moles\cdot cm^{-1}L^{-1}$	$\varepsilon_{Hb}/moles\cdot cm^{-1}L^{-1}$
617 nm	1068	6927.2
850 nm	1058	7136

## References

[1] Totty J. P. Moss J. W. E. Barker E. et al., “The impact of surgical site infection on hospitalisation, treatment costs, and health-related quality of life after vascular surgery,” Int. Wound J. 18(3), 261–268 (2021). 10.1111/iwj.13526 33331066 PMC8243999

[2] Petherick E. S. Dalton J. E. Moore P. J. et al., “Methods for identifying surgical wound infection after discharge from hospital: A systematic review,” BMC Infect. Dis. 6(1), 170 (2006). 10.1186/1471-2334-6-170 17129368 PMC1697816

[3] Cutting K. Harding K., “Criteria for identifying wound infection,” J. Wound Care 3(4), 198–201 (1994). 10.12968/jowc.1994.3.4.198 27922298

[4] Cutting K. F. White R., “Defined and refined: criteria for identifying wound infection revisited,” Br. J. Community Nurs. 9(Sup1), S6–S15 (2004). 10.12968/bjcn.2004.9.Sup1.12495 15029002

[5] Li S. Renick P. Senkowsky J. et al., “Diagnostics for wound infections,” Adv. Wound Care 10(6), 317–327 (2021). 10.1089/wound.2019.1103 PMC8082727 32496977

[6] Smith M. E. Robinowitz N. Chaulk P. et al., “Comparison of chronic wound culture techniques: Swab versus curetted tissue for microbial recovery,” Br. J. Community Nurs. 19(Sup9), S22–S26 (2014). 10.12968/bjcn.2014.19.Sup9.S22 25192558 PMC4267254

[7] Bonham P. A., “Swab cultures for diagnosing wound infections: A literature review and clinical guideline,” J. Wound Ostomy Cont. Nurs. 36(4), 389–395 (2009). 10.1097/WON.0b013e3181aaef7f 19609159

[8] Jayachandran M. Rodriguez S. Solis E. et al., “Critical Review of Noninvasive Optical Technologies for Wound Imaging,” Adv. Wound Care 5(8), 349–359 (2016). 10.1089/wound.2015.0678 PMC4991615 27602254

[9] Huang D. Swanson E. A. Lin C. P. et al., “Optical coherence tomography,” Science 254(5035), 1178–1181 (1991). 10.1126/science.1957169 1957169 PMC4638169

[10] Srinivas S. M. de Boer J. F. Park H. et al., “Determination of burn depth by polarization-sensitive optical coherence tomography,” J. Biomed. Opt. 9(1), 207–212 (2004). 10.1117/1.1629680 14715075

[11] Park K. S. Choi W. J. Song S. et al., “Multifunctional in vivo imaging for monitoring wound healing using swept-source polarization-sensitive optical coherence tomography,” Lasers Surg. Med. 50(3), 213–221 (2018). 10.1002/lsm.22767 29193202 PMC5867210

[12] Singer A. J. Wang Z. McClain S. A. et al., “Optical coherence tomography: a noninvasive method to assess wound reepithelialization,” Acad. Emerg. Med. 14(5), 387–391 (2007). 10.1197/j.aem.2006.11.022 17363766

[13] Cobb M. J. Chen Y. Underwood R. A. et al., “Noninvasive assessment of cutaneous wound healing using ultrahigh-resolution optical coherence tomography,” J. Biomed. Opt. 11(6), 064002 (2006). 10.1117/1.2388152 17212525

[14] Pierce M. C. Sheridan R. L. Park B. H. et al., “Collagen denaturation can be quantified in burned human skin using polarization-sensitive optical coherence tomography,” Burns 30(6), 511–517 (2004). 10.1016/j.burns.2004.02.004 15302415

[15] Saiko G. Lombardi P. Au Y. et al., “Hyperspectral imaging in wound care: a systematic review,” Int. Wound J. 17(6), 1840–1856 (2020). 10.1111/iwj.13474 32830443 PMC7949456

[16] Stamatas G. N. Balas C. J. Kollias N., “Hyperspectral image acquisition and analysis of skin,” in *Spectral Imaging: Instrumentation, Applications, and Analysis II* , vol. 4959 (SPIE, 2003), pp. 77–82.

[17] Dicker D. T. Lerner J. Van Belle P. et al., “Differentiation of normal skin and melanoma using high resolution hyperspectral imaging,” Cancer biology therapy 5(8), 1033–1038 (2006). 10.4161/cbt.5.8.3261 16931902

[18] Calin M. A. Coman T. Parasca S. V. et al., “Hyperspectral imaging-based wound analysis using mixture-tuned matched filtering classification method,” J. Biomed. Opt. 20(4), 046004 (2015). 10.1117/1.JBO.20.4.046004 25867619

[19] Yudovsky D. Pilon L., “Rapid and accurate estimation of blood saturation, melanin content, and epidermis thickness from spectral diffuse reflectance,” Appl. Opt. 49(10), 1707–1719 (2010). 10.1364/AO.49.001707 20357850

[20] Khaodhiar L. Dinh T. Schomacker K. T. et al., “The use of medical hyperspectral technology to evaluate microcirculatory changes in diabetic foot ulcers and to predict clinical outcomes,” Diabetes Care 30(4), 903–910 (2007). 10.2337/dc06-2209 17303790

[21] Calin M. A. Parasca S. V. Savastru R. et al., “Characterization of burns using hyperspectral imaging technique–a preliminary study,” Burns 41(1), 118–124 (2015). 10.1016/j.burns.2014.05.002 24997530

[22] Zuzak K. J. Schaeberle M. D. Lewis E. N. et al., “Visible reflectance hyperspectral imaging: characterization of a noninvasive, in vivo system for determining tissue perfusion,” Anal. Chem. 74(9), 2021–2028 (2002). 10.1021/ac011275f 12033302

[23] Wild T. Becker M. Winter J. et al., “Hyperspectral imaging of tissue perfusion and oxygenation in wounds: assessing the impact of a micro capillary dressing,” J. Wound Care 27(1), 38–51 (2018). 10.12968/jowc.2018.27.1.38 29333931

[24] Dietrich M. Marx S. von der Forst M. et al., “Hyperspectral imaging for perioperative monitoring of microcirculatory tissue oxygenation and tissue water content in pancreatic surgery–an observational clinical pilot study,” Perioper Med. 10(1), 42 (2021). 10.1186/s13741-021-00211-6 PMC8638177 34847953

[25] Hao S. Xiong Y. Guo S. et al., “Development and performance validation of a low-cost algorithms-based hyperspectral imaging system for radiodermatitis assessment,” Biomed. Opt. Express 14(9), 4990 (2023). 10.1364/BOE.500067 37791251 PMC10545207

[26] Stern M. D., “In vivo evaluation of microcirculation by coherent light scattering,” Nature 254(5495), 56–58 (1975). 10.1038/254056a0 1113878

[27] Essex T. Byrne P., “A laser doppler scanner for imaging blood flow in skin,” J. Biomed. Eng. 13(3), 189–194 (1991). 10.1016/0141-5425(91)90125-Q 1870327

[28] Hoeksema H. Van de Sijpe K. Tondu T. et al., “Accuracy of early burn depth assessment by laser doppler imaging on different days post burn,” Burns 35(1), 36–45 (2009). 10.1016/j.burns.2008.08.011 18952377

[29] Holland A. Martin H. Cass D., “Laser doppler imaging prediction of burn wound outcome in children,” Burns 28(1), 11–17 (2002). 10.1016/S0305-4179(01)00064-X 11834324

[30] Beard P., “Biomedical photoacoustic imaging,” Interface focus 1(4), 602–631 (2011). 10.1098/rsfs.2011.0028 22866233 PMC3262268

[31] Deán-Ben X. L. Razansky D., “Optoacoustic imaging of the skin,” Exp. Dermatol. 30(11), 1598–1609 (2021). 10.1111/exd.14386 33987867

[32] Tsunoi Y. Sato S. Kawauchi S. et al., “In vivo photoacoustic molecular imaging of the distribution of serum albumin in rat burned skin,” Burns 39(7), 1403–1408 (2013). 10.1016/j.burns.2013.03.007 23597848

[33] Zhang H. F. Maslov K. Stoica G. et al., “Imaging acute thermal burns by photoacoustic microscopy,” J. Biomed. Opt. 11(5), 054033 (2006). 10.1117/1.2355667 17092182

[34] Vionnet L. Gateau J. Schwarz M. et al., “24-mhz scanner for optoacoustic imaging of skin and burn,” IEEE Trans. Med. Imaging 33(2), 535–545 (2013). 10.1109/TMI.2013.2289930 24216682

[35] Hariri A. Chen F. Moore C. et al., “Noninvasive staging of pressure ulcers using photoacoustic imaging,” Wound Repair and Regeneration 27(5), 488–496 (2019). 10.1111/wrr.12751 31301258 PMC8043767

[36] Harary T. Nagli M. Suleymanov N. et al., “Large-field-of-view optical-resolution optoacoustic microscopy using a stationary silicon-photonics acoustic detector,” J. Biomed. Opt. 29(S1), 1–11 (2024). 10.1117/1.JBO.29.S1.S11511 PMC10768684 38187934

[37] Cuccia D. J. Bevilacqua F. Durkin A. J. et al., “Quantitation and mapping of tissue optical properties using modulated imaging,” J. Biomed. Opt. 14(2), 024012 (2009). 10.1117/1.3088140 19405742 PMC2868524

[38] Gioux S. Mazhar A. Cuccia D. J., “Spatial frequency domain imaging in 2019: principles, applications, and perspectives,” J. Biomed. Opt. 18, 035001 (2019). 10.1117/1.JBO.24.7.071613 PMC6995958 31222987

[39] Mazhar A. Saggese S. Pollins A. C. et al., “Noncontact imaging of burn depth and extent in a porcine model using spatial frequency domain imaging,” J. Biomed. Opt. 19(8), 086019 (2014). 10.1117/1.JBO.19.8.086019 25147961 PMC4141219

[40] Nguyen J. Q. Crouzet C. Mai T. et al., “Spatial frequency domain imaging of burn wounds in a preclinical model of graded burn severity,” J. Biomed. Opt. 18(6), 066010 (2013). 10.1117/1.JBO.18.6.066010 23764696 PMC3680730

[41] Nguyen T. T. Ramella-Roman J. C. Moffatt L. T. et al., “Novel application of a spatial frequency domain imaging system to determine signature spectral differences between infected and noninfected burn wounds,” J. Burn. Care Res. 34(1), 44–50 (2013). 10.1097/BCR.0b013e318269be30 23292572 PMC3539220

[42] Weinkauf C. Mazhar A. Vaishnav K. et al., “Near-instant noninvasive optical imaging of tissue perfusion for vascular assessment,” Journal of Vascular Surgery 69(2), 555–562 (2019). 10.1016/j.jvs.2018.06.202 30292608

[43] Yafi A. Muakkassa F. K. Pasupneti T. et al., “Quantitative skin assessment using spatial frequency domain imaging (SFDI) in patients with or at high risk for pressure ulcers,” Lasers Surg. Med. 49(9), 827–834 (2017). 10.1002/lsm.22692 28586092

[44] Pilvar A. Mehendale A. M. Karrobi K. et al., “Spatial frequency domain imaging for the assessment of scleroderma skin involvement,” Biomed. Opt. Express 14(6), 2955 (2023). 10.1364/BOE.489609 37342706 PMC10278615

[45] Applegate M. B. Karrobi K. Angelo J. P. et al., “OpenSFDI: an open-source guide for constructing a spatial frequency domain imaging system,” J. Biomed. Opt. 25(01), 1 (2020). 10.1117/1.JBO.25.1.016002 PMC7008504 31925946

[46] Bounds A. D. Girkin J. M., “Early stage dental caries detection using near infrared spatial frequency domain imaging,” Sci. Rep. 11(1), 2433 (2021). 10.1038/s41598-021-81872-7 33510285 PMC7844280

[47] Crowley J. Gordon G. S. D., “Ultra-miniature dual-wavelength spatial frequency domain imaging for micro-endoscopy,” J. Biomed. Opt. 29(02), 026002 (2024). 10.1117/1.JBO.29.2.026002 38312854 PMC10832795

[48] Laughney A. M. Krishnaswamy V. Rizzo E. J. et al., “Spectral discrimination of breast pathologies in situ using spatial frequency domain imaging,” Breast Cancer Res. 15(4), R61 (2013). 10.1186/bcr3455 23915805 PMC3979079

[49] Wirth D. Sibai M. Olson J. et al., “Feasibility of using spatial frequency-domain imaging intraoperatively during tumor resection,” J. Biomed. Opt. 24(07), 1 (2018). 10.1117/1.JBO.24.7.071608 PMC6995878 30378351

[50] Wirth D. J. Sibai M. Wilson B. C. et al., “First experience with spatial frequency domain imaging and red-lightexcitation of protoporphyrin ix fluorescence during tumorresection,” Biomed. Opt. Express 11(8), 4306–4315 (2020). 10.1364/BOE.397507 32923044 PMC7449712

[51] Zhang L. Bounds A. Girkin J., “Monte Carlo simulations and phantom modeling for spatial frequency domain imaging of surgical wound monitoring,” J. Biomed. Opt. 28(12), 126003 (2023). 10.1117/1.JBO.28.12.126003 38098981 PMC10720737

[52] Zhang L. Bounds A. Fleming J. et al., “Monitoring of surgical wound healing using spatial frequency domain imaging,” in *Biomedical Applications of Light Scattering XII* , vol. 11974 Wax A. Backman V., eds., International Society for Optics and Photonics (SPIE, 2022), p. 1197407.

[53] Zhang L. Bounds A. D. Fleming J. P. et al., “Spatial frequency domain imaging (sfdi) method for characterizing surgical wound sites,” in Imaging and Applied Optics Congress 2022 (3D, AOA, COSI, ISA, pcAOP), (Optica Publishing Group, 2022), p. ITu3E.2.

[54] Zhang L. Bounds A. D. Fleming J. P. et al., “Characterizing surgical wound sites with spatial frequency domain imaging (sfdi),” in Latin America Optics and Photonics (LAOP) Conference 2022, (Optica Publishing Group, 2022), p. Tu1B.7.

[55] Eming S. A. Martin P. Tomic-Canic M., “Wound repair and regeneration: Mechanisms, signaling, and translation,” Sci. Transl. Med. 6(265), 1 (2014). 10.1126/scitranslmed.3009337 PMC4973620 25473038

[56] Velnar T. Bailey T. Smrkolj V., “The wound healing process: An overview of the cellular and molecular mechanisms,” J. Int. Med. Res. 37(5), 1528–1542 (2009). 10.1177/147323000903700531 19930861

[57] Kanzler M. H. Gorsulowsky D. C. a. Swanson N., “Basic Mechanisms in the Healing,” The Journal of dermatologic surgery and oncology 12(11), 1156–1164 (1986). 10.1111/j.1524-4725.1986.tb02099.x 3490500

[58] Schultz G. S. Chin G. A. Diegelmann R. F., “Principles of Wound Healing,” (2011). 30485016

[59] Eming S. A. Krieg T. Davidson J. M., “Inflammation in wound repair: Molecular and cellular mechanisms,” J. Invest. Dermatol. 127(3), 514–525 (2007). 10.1038/sj.jid.5700701 17299434

[60] Hunt M. Torres M. Bachar-Wikström E. et al., “Multifaceted roles of mitochondria in wound healing and chronic wound pathogenesis,” Front. Cell Dev. Biol. 11, 1252318 (2023). 10.3389/fcell.2023.1252318 37771375 PMC10523588

[61] Shirakata Y. Kimura R. Nanba D. et al., “Heparin-binding EGF-like growth factor accelerates keratinocyte migration and skin wound healing,” J. Cell Sci. 118(11), 2363–2370 (2005). 10.1242/jcs.02346 15923649

[62] Mathew-Steiner S. S. Roy S. Sen C. K., “Collagen in wound healing,” Bioengineering 8(5), 63 (2021). 10.3390/bioengineering8050063 34064689 PMC8151502

[63] Wallace H. A. Zito P. M., “Wound Healing Phases,” (2019). 29262065

[64] Landén N. X. Li D. Ståhle M., “Transition from inflammation to proliferation: a critical step during wound healing,” Cell. Mol. Life Sci. 73(20), 3861–3885 (2016). 10.1007/s00018-016-2268-0 27180275 PMC5021733

[65] Omar Skalli; Giulio Gabbiani, *The Biology of the Myofibroblast Relationship to Wound Contraction and Fibrocontractive Diseases*, vol. 69 (1998).

[66] DiPietro L. A., “Angiogenesis and wound repair: when enough is enough,” Journal of Leucocyte Biology 100(5), 979–984 (2016). 10.1189/jlb.4MR0316-102R PMC6608066 27406995

[67] Clark R. A., “Regulation of fibroplasia in cutaneous wound repair,” American Journal of the Medical Sciences 306(1), 42–48 (1993). 10.1097/00000441-199307000-00011 8328509

[68] Singh D. Rai V. Agrawal D. K., “Regulation of collagen i and collagen iii in tissue injury and regeneration,” Cardiol. Cardiovasc. Med. 07(01), 5 (2023). 10.26502/fccm.92920302 PMC9912297 36776717

[69] Cheung S. S., “Responses of the hands and feet to cold exposure,” Temperature 2(1), 105–120 (2015). 10.1080/23328940.2015.1008890 PMC4843861 27227009

[70] Lister T. Wright P. A. Chappell P. H., “Optical properties of human skin,” J. Biomed. Opt. 17(9), 0909011 (2012). 10.1117/1.JBO.17.9.090901 23085902

[71] Lowe D. G., “Distinctive image features from scale-invariant keypoints,” International Journal of Computer Vision 60(2), 91–110 (2004). 10.1023/B:VISI.0000029664.99615.94

[72] Fischler M. A. Bolles R. C., “Random sample consensus: A Paradigm for Model Fitting with Applications to Image Analysis and Automated Cartography,” Commun. ACM 24(6), 381–395 (1981). 10.1145/358669.358692

[73] “appSFDI,” (2019).

[74] Ponticorvo A. Burmeister D. M. Yang B. et al., “Quantitative assessment of graded burn wounds in a porcine model using spatial frequency domain imaging (sfdi) and laser speckle imaging (lsi),” Biomed. Opt. Express 5(10), 3467–3481 (2014). 10.1364/BOE.5.003467 25360365 PMC4206317

[75] Kennedy G. T. Stone R. Kowalczewski A. C. et al., “Spatial frequency domain imaging: a quantitative, noninvasive tool for in vivo monitoring of burn wound and skin graft healing,” J. Biomed. Opt. 24(07), 1 (2019). 10.1117/1.JBO.24.7.071615 PMC6630099 31313538

[76] Schreml S. Szeimies R. M. Prantl L. et al., “Oxygen in acute and chronic wound healing,” Br. J. Dermatol. 163(2), 257–268 (2010). 10.1111/j.1365-2133.2010.09804.x 20394633

[77] Castilla D. M. Liu Z.-J. Velazquez O. C., “Oxygen: Implications for Wound Healing,” Adv. Wound Care 1(6), 225–230 (2012). 10.1089/wound.2011.0319 PMC3625368 24527310

[78] Ponticorvo A. Rowland R. Baldado M. et al., “Spatial Frequency Domain Imaging (SFDI) of clinical burns: A case report,” Burns Open 4(2), 67–71 (2020). 10.1016/j.burnso.2020.02.004 32832745 PMC7442210

[79] Soret M. Bacharach S. L. Buvat I., “Partial-Volume Effect in PET Tumor Imaging,” J. Nucl. Med. 48(6), 932–945 (2007). 10.2967/jnumed.106.035774 17504879

[80] Ghassemi P. Shupp J. W. Moffatt L. T. et al., “A novel spectral imaging system for quantitative analysis of hypertrophic scar,” Photonic Therapeutics and Diagnostics IX 8565, 85650U (2013). 10.1117/12.2006096

[81] Ghassemi P. Travis T. E. Moffatt L. T. et al., “A polarized multispectral imaging system for quantitative assessment of hypertrophic scars,” Biomed. Opt. Express 5(10), 3337 (2014). 10.1364/BOE.5.003337 25360354 PMC4206306

[82] Weingarten M. S. Neidrauer M. Mateo A. et al., “Prediction of wound healing in human diabetic foot ulcers by diffuse near-infrared spectroscopy: A pilot study,” Wound Repair and Regeneration 18(2), 180–185 (2010). 10.1111/j.1524-475X.2010.00583.x 20419875

[83] Walling P. L. Dabney J. M., “Moisture in skin by near-infrared reflectance spectroscopy,” J. Soc. Cosmet. Chem 40, 151–171 (1989).

[84] Graham C. Girkin J. M. Bourgenot C., “Freeform based hyperspectral imager for moisture sensing (fymos),” Opt. Express 29(11), 16007–16018 (2021). 10.1364/OE.425660 34154173

[85] Micheels J. Aisbjorn B. Sorensen B., “Laser doppler flowmetry. a new non-invasive measurement of microcirculation in intensive care?” Resuscitation 12(1), 31–39 (1984). 10.1016/0300-9572(84)90056-X 6330823

[86] Ishii T. Takabe S. Yanagawa Y. et al., “Laser doppler blood flowmeter as a useful instrument for the early detection of lower extremity peripheral arterial disease in hemodialysis patients: an observational study,” BMC Nephrol. 20(1), 470 (2019). 10.1186/s12882-019-1653-y 31852449 PMC6921472

[87] Prahl S., “Tabulated molar extinction coefficient for hemoglobin in water,” https://omlc.org/spectra/hemoglobin/summary.html (1998).

